# Cancer and occupational exposure to pesticides: an umbrella review

**DOI:** 10.1007/s00420-020-01638-y

**Published:** 2021-01-25

**Authors:** Carol J. Burns, Daland R. Juberg

**Affiliations:** 1 Sanford, USA; 2 Indianapolis, USA

**Keywords:** Pesticides, Epidemiology, Cancer, Farming, Toxicology

## Abstract

**Purpose:**

The aim was to identify the scope of the epidemiology literature reviewed regarding the risk of cancer as related to occupational exposure to pesticides and to compare regulatory toxicity results where feasible.

**Methods:**

Review studies of breast, lung, prostate, non-Hodgkin lymphoma, and colorectal cancer were identified from the published literature from 2010 to 2020 using a priori inclusion and exclusion criteria. Epidemiology observations were first assessed and then compared against carcinogenicity profiles derived from regulatory toxicology studies.

**Results:**

Several active ingredients were associated with specific cancer but overall, there was neither strong nor consistent epidemiologic data supportive of a positive association between pesticide exposure in occupational settings and cancer. Authors noted common themes related to the heterogeneity of exposure, study design, control for confounders, and the challenge to collect these data reliably and validly with an adequate sample size. Toxicology studies in laboratory animals that assessed carcinogenic potential did not reveal cancer outcomes that were concordant with reported epidemiologic findings.

**Conclusions:**

Farming and pesticides represent diverse exposures that are difficult to quantify in epidemiologic studies. Going forward, investigators will need creative and novel approaches for exposure assessment. Integration of epidemiologic and toxicological studies with attention to biological plausibility, mode of toxicological action and relevance to humans will increase the ability to better assess associations between pesticides and cancer.

**Supplementary Information:**

The online version contains supplementary material available at 10.1007/s00420-020-01638-y.

## Introduction

Cancer etiology has been studied for decades to characterize risk factors such as genetic predisposition, lifestyle, and environmental factors, the latter with particular focus on chemical agents, including pesticides. Generally recognized modifiable risk factors for cancer are related to behavior and diet, including smoking, alcohol consumption, unhealthy diet, obesity, insufficient physical activity, and certain infections (World Health Organization 2018). Given the prevalence and use of agricultural chemicals in farming, it is important to seek clarity on whether occupational exposure to pesticides is an independent risk factor for cancer. Because pesticides are evaluated for carcinogenicity in laboratory animals as a part of regulatory requirements, it is also relevant to understand the outcomes of this testing and how it may inform putative associations between cancer and pesticides in humans. These studies are conducted not only for evaluation of intrinsic carcinogenic potential in an animal model but also for determination of relevance to humans and cancer classification and labeling purposes.

The U.S. National Cancer Institute (NCI) and the European Food Safety Authority (EFSA) published reviews of the cancer burden related to pesticide exposure in 2012 and 2013 (Alavanja and Bonner 2012; Alavanja et al. 2013; Ntzani et al. 2013). The EFSA-sponsored reviewers noted that while there were many epidemiologic studies, there were limitations to drawing conclusions (Ntzani et al. 2013). The NCI reviewers suggested “substantial risks” and recommended multidisciplinary efforts to improve the scientific understanding (Alavanja and Bonner 2012; Alavanja et al. 2013). Evaluations of selected active ingredients were also recently evaluated and reported by the International Agency for Research on Cancer (IARC) (2017, 2018,  2019). Lindane was the only pesticide determined to be carcinogenic based upon *sufficient* data in both humans and animals. The epidemiology evidence for other pesticides reviewed was considered to be limited or inadequate.

To provide a more multidisciplinary view, the aim of this review was to identify the scope of the epidemiology literature reviewed, i.e. a review of reviews, in the past decade regarding the risk of cancer as related to occupational exposure to pesticides and to compare the conclusions from regulatory cancer bioassays in animals where feasible for pesticides and cancer. The objectives were to evaluate: (1) the evidence within epidemiologic literature of farmers and other agricultural workers exposed to pesticides and the association with cancer of the lung, breast, colorectum, prostate, and non-Hodgkin lymphoma (NHL) and (2) whether there is consistent and supportive evidence and biological plausibility for these cancer types from animal toxicology studies.

## Methods

A review of qualitative and quantitative reviews was undertaken following the recommendations of Aromataris et al. (2015) and with consideration for systematic review evaluation in general (Shea et al. 2017; Moher et al. 2009). Each step of the process was performed independently by both authors, with stepwise discussion to clarify and resolve any disagreements.

The literature search was focused upon reviews of epidemiologic studies of globally prevalent cancers of lung, prostate, colorectal, breast and NHL and occupational exposure to pesticides published in the last 10 years (e.g. (Arnold et al. 2015; Blair et al. 1992; IARC 2020b)). This time period was selected to bound the reviewable literature from the 2012 and 2013 reviews (Alavanja and Bonner 2012; Alavanja et al. 2013; Ntzani et al. 2013) and to capture recently reviewed studies. PubMed and Web of Science databases were searched in January 2020. The search strategy was: (cancer or neoplasm or tumor) and human and (pesticide or herbicide or insecticide or fungicide or farm) and (meta-analysis or systematic review or review), allowing for truncation of words and MESH terms. Both searches were limited to peer reviewed English-language publications from 2010 to 2020. The search was not restricted to study design, cancer mortality or incidence, or use of systematic approaches. Editorials were not included. Also excluded were reviews specific to other cancers, children, general populations, a focus upon exposure without health endpoints and studies of animals. Reviews of dioxins were excluded as they are contaminants and not a specific active ingredient.

**Fig. 1:**
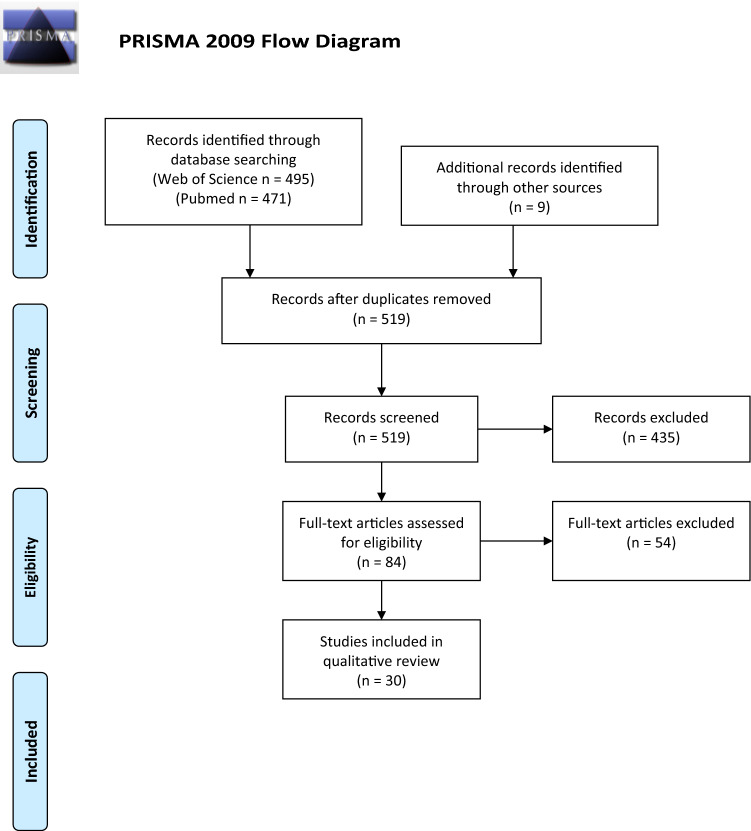
PRISMA 2009 flow diagram

The IARC monographs from this period were used to identify published meta-analyses of one or more pesticides. The PRISMA flowchart is shown in Fig. 1. After removing duplicates, a total of 519 publications were identified. Screening based on titles and abstracts narrowed the number of reviews to 84 publications based upon the criteria above. The full text of these 84 studies was reviewed by both authors and attenuated to 30 primary publications, excluding editorials, publications on other cancers or not of epidemiology. Aspects of each study were recorded. These included stated study objectives, databases searched, data range of search, inclusion and exclusion criteria provided, number of studies reviewed, number of studies that were from a single source (Agricultural Health Study, AHS), instrument(s) used to assess quality, number of studies that were occupational, and approaches to the weight of evidence interpretation.

## Results: epidemiology

We identified 30 review publications that met the inclusion criteria (Table 1). More than half of the reviews focused upon one type of cancer and many concentrated upon a specific pesticide. The number of reviewed studies was reflective of the scope of the review. For example, reviewers evaluated more than 100 studies in two publications of all cancers (Alavanja and Bonner 2012; Ntzani et al. 2013) while there were only five studies included in the review of methyl bromide and prostate cancer (Budnik et al. 2012). Most reviewers provided information consistent with systematic review processes such as databases searched, data range of the search, and inclusion and exclusion criteria, with a few exceptions (Alavanja et al. 2013; Boffetta et al. 2013; Jowa and Howd 2011). The number of AHS studies per review reflects the considerable number of publications from this single cohort study of farmer applicators in the United States. Approximately half of the reviewers incorporated a quality assessment of each reviewed study. Many used the Newcastle Ottawa Scale (NOS), two used the Effective Public Health Practice Project (EPHPP), while others evaluated study quality using their own list of elements.

**Table 1:** Reviews of cancer and pesticides

First author (Year)	Cancer type	Exposure	N Search databases	Inclusion/ exclusion criteria	Date range	N studies	N of AHS	Quality instrument
Alavanja (2013)	All	Pesticides	NR	NR	NR	NR	NR	None
Alavanja (2012)	All	Pesticides	1	Y	1990–2010	103*	NR	None
Boffetta (2013)	All	Atrazine	NR	NR	NR	NR	NR	None
Boffetta (2018)	All	Permethrin	3	Y	x-2017	18	11	None
Burns (2012)	All	2,4-D	> 3	Y	2000–2012	27*	0	None
Jowa (2011)	All	Atrazine (& related chlorotriazines)	NR	NR	NR	25*	2	None
Mink (2012)	All	Glyphosate	3	NR	NR	21	7	None
Nguyen (2018)	All	Pesticides	> 3	Y	1990—2015	5	5	EPHPP
Ntzani (2013)	All	Pesticides	> 3	Y	2006—2012	164	30	Own
Sathiakumar (2011)	All	Triazine herbicides	3	NR	NR	36	4	Own
Von Stackelberg (2013)	All	2,4-D, MCPA	3	NR	NR	41*	0	None
Alexander (2012)	Colorectal	Pesticides	1	Y	x-2010	42	29	Own
Oddone (2014)	Colorectal	Pesticides	2	Y	1960–2013	83 (12 of farming)	NR	None
Acquavella (2016)	NHL	Glyphosate	2	Y	x – 2015	16	1	Own
Chang (2016)	NHL	Glyphosate	3	Y	NR	12	1	Own
Goodman (2015)	NHL, Prostate	2,4-D	3	Y	x- 2014	11	0	Own
Goodman (2017)	NHL	2,4-D	Update to 2015	Per Goodman 2015	Per Goodman 2015	10	1	Own
Hu (2017)	NHL	Organophosphates	2	Y	1985–2017	10	5	NOS
Jayakody (2015)	NHL	Phenoxy	2	NR	x – 2014	43	0	None
Schinasi (2014)	NHL	Pesticides	2	Y	1980–2014	44	16	None
Smith (2017)	NHL	2,4-D	3	Y	x—2016	12	0	NOS
Zhang (2019)	NHL	Glyphosate	> 3	Y	NR	7	2	NOS
Budnik (2012)	Prostate	Methyl bromide	1	Y	1990–2011	5	2	None
Depczynski (2014)	Prostate	Pesticides, farming	> 3	Y	2002–2013	18	1	EPHPP
Doolan (2014)	Prostate	Pesticides, farming	2	NR	2000–2012	28*	NR	None
Krstev (2019)	Prostate	Pesticides, farming	2	Y	1966—2015	18 pesticides, 26 farming	1	None
Lewis-Mikhael (2016)	Prostate	Pesticides	3	Y	1985–2014	52	21	NOS
Lewis-Mikhael (2015)	Prostate	Organochlorine	3	Y	x – 2015	15	1	NOS
Ragin (2013)	Prostate	Farming	1	Y	x – 2012	12	0	None
Silva (2016)	Prostate	Pesticides	3	Y	x – 2015	49	29	NOS

*NR* Not reported, *NHL* Non-Hodgkin Lymphoma, *NOS* Newcastle Ottawa Scale, *EPHPP* effective public health practice project, *Based on a count of the studies, number not reported by the authors

## Breast, lung and colorectal cancers

There were few reviews that addressed breast, lung and/or colorectal cancers and all were qualitative, i.e. narrative reviews. Ntzani et al. (2013) discussed epidemiologic study results for breast, colorectal, and lung cancers, concluding that for the most part, the evidence was limited and inconclusive. Boffetta et al. also discussed a lack of association with pesticide exposure and breast cancer in the reviews of permethrin (Boffetta and Desai 2018) and atrazine (Boffetta et al. 2013). Two reviews focused upon colorectal cancer with neither indicating an increased risk for any pesticide (Alexander et al. 2012; Oddone et al. 2014). In their reviews of triazine herbicides, Jowa and Howd characterized the epidemiologic data as being inadequate for cancers of breast and colon (Jowa and Howd 2011). Sathiakumar et al. discussed results of a single case-control study of colon cancer for which odds ratios differed by comparison group (OR = 1.4 compared with nonfarmers and OR = 0.6 compared with farmers who had not used triazines) (Sathiakumar et al. 2011).

## Prostate cancer

Fourteen reviews assessed the association of prostate cancer and exposure to pesticides and/or farming. As shown in Table A1 in the Appendix these publications varied in their focus (general vs. specific pesticides) and approach (qualitative vs. quantitative). The reviewers of several specific active ingredients (i.e., 2,4-D, atrazine, and organophosphates) reported no significant associations between exposure and prostate cancer (Boffetta et al. 2013; Goodman et al. 2015; Jowa and Howd 2011; Krstev and Knutsson 2019), while the review of methyl bromide was suggestive of an association based on a meta-analysis of three studies (meta Odds Ratio, mOR 1.21; 95% CI 0.98–1.49) (Budnik et al. 2012). The association of organochlorines as a group and individually was evaluated by several reviewers (Krstev and Knutsson 2019; Lewis-Mikhael et al. 2015, 2016; Ntzani et al. 2013). Statistically significant associations were reported for organochlorines, collectively, based on 17 studies in Krestev et al. (2019) (meta Relative Risk, mRR 1.08, 95% CI 1.03–1.14,) and eight studies in Lewis-Mikhael et al. (2016) (mRR = 1.35, 95% CI 1.02–1.67).

Several reviewers evaluated studies with nonspecific exposure to pesticides and/or farming. Authors noted common themes related to the heterogeneity of exposure, outcome (i.e. mortality vs. incidence), study design, and control for confounders (Depczynski and Lower 2014; Doolan et al. 2014; Lewis-Mikhael et al. 2016; Silva et al. 2016). The reviewers Lewis-Mikhael and colleagues evaluated 52 studies and stratified pooled estimates based on different quality-related aspects (Lewis-Mikhael et al. 2016). From this, the authors suggested that biases of exposure misclassification, using hospital controls and not controlling for family history of prostate cancer, may have led to higher risk estimates in selected studies. For example, in analyses stratified by exposure assessment quality (using the NOS), lower meta odds ratios were reported for studies with high quality (mOR 0.85; 95% CI 0.57 – 1.14) compared to studies with low quality (mOR 2.19; 95% CI 1.38–3.00).

Another large systematic review by Silva et al. (2016) used the NOS tool to exclude one study for low quality. However, there was no further evaluation or discussion on aspects related to quality or bias. The authors pointed to the diversity in study populations, methodologies, and the putative role of family history of prostate cancer. Ntzani et al. (2013) approached their review by discussing the results of the AHS publications (*N* = 25, 65% of the reviewed studies) for which the analyses were for specific pesticides. The discussion on the other studies pointed to observations of increased risk and for which study quality was moderate or low.

Several reviewers summarized the study finding based on occupation (farming/farm workers). Krestev and Knutsson reported a null meta relative risk (mRR 0.99; 95% CI 0.95–1.02) (Krestev and Knutsson 2019) and Depczynski and Lower described the risk of prostate cancer among farmers as weak with many methodological inconsistencies (Depczynski and Lower 2014). In contrast, Ragin et al. (2013) reported a higher risk of prostate cancer among farmers, but an inverse relation for pesticide use (mOR 0.68; 95% 0.49 – 0.96).

## Non-Hodgkin lymphoma (NHL)

The reviews with a focus on NHL and the authors’ conclusions are shown in Table A2 in the Appendix. Similar to the reviews of prostate cancer, about half discussed the literature qualitatively while others used a quantitative approach of meta-analyses. There are research challenges related to the evolution of the diagnostic definition of lymphohematopoietic cancers, and NHL specifically. Several reviewers discussed these difficulties and the impact that reduced statistical power to study histological subtypes can present for epidemiologic research (e.g. (Alavanja et al. 2013; Schinasi and Leon 2014; von Stackelberg 2013)).

Alavanja et al. (2013) acknowledged that their review was not comprehensive and as such, only highlighted some inconsistencies in the literature. Ntzani et al. (2013) and Schinasi and Leon (2014) were more exhaustive on the number of studies identified and each took different approaches to evaluate the literature. Ntzani et al. (2013) discussed many results in the context of the AHS and did not focus on any specific pesticide. In contrast, Schinasi and Leon were quantitative in their review, stratifying their pooled analyses and discussion on specific active ingredients as well as providing discussion by gender, design, diagnosis period, and geographic area (Schinasi and Leon 2014).

Hu et al. and Schinasi and Leon, reported statistically significant associations with diazinon and NHL based upon seven and three studies, respectively (Hu et al. 2017; Schinasi and Leon 2014). Several reviews provided results on specific herbicides, such as atrazine (class triazine), with mixed conclusions (Boffetta et al. 2013; Sathiakumar et al. 2011; Schinasi and Leon 2014). For example, Schinasi and Leon, reported a significant mRR of 1.5 for triazine exposure (Schinasi and Leon 2014) while the larger narrative style reviews by Boffetta et al. and Sathiakumar et al. reported a lack of a consistent association (Boffetta et al. 2013; Sathiakumar et al. 2011).

### NHL reviews of 2,4-D

The authors of three narrative style reviews of phenoxy herbicide 2,4-D and NHL discussed inconsistencies of results across studies (Burns and Swaen 2012; Jayakody et al. 2015; von Stackelberg 2013). Jayakody et al. (2015) highlighted the challenges inherent in evaluating uncommon exposures and uncommon outcomes. Von Stackelberg (2013) pointed to the concomitant use of different herbicides among applicators and farmers, while Burns and Swaen (2012) compared studies by statistical significance, evidence of a dose-response, and consistency within and across studies.

Four other publications summarized the 2,4-D literature quantitatively, i.e. meta-analyses. As shown in Table A2, Schinasi and Leon (2014) and Smith et al. (2017) reported similar meta estimates of 1.4 and 1.3 for NHL and ever use of 2,4-D. In contrast, Goodman et al. (2015, 2017) reported no increased risk of 2,4-D and NHL among nine studies (mRR = 0.97, 95% CI 0.77–1.22), nor when including unpublished AHS results (mRR 0.97, 95% CI 0.79–1.18). These publications used different selection criteria that likely impacted the pooled result. For example, the meta-analyses by Smith and colleagues (2017) included two studies with specific diagnoses often excluded from reviews of NHL, B cell lymphoma, and hairy cell leukemia (Cocco et al. 2013; Nordstrom et al. 1998). Goodman et al. (2015) used the results from a 2011 publication from a Canadian study (RR = 0.94) while Smith et al. (2017) elected to use the 2001 results from the same study (RR = 1.3).

### NHL reviews of glyphosate

There were four reviews of glyphosate and NHL studies in addition to the glyphosate-specific analyses of Schinasi and Leon (2014). Mink et al. (2012) discussed the limitations of self-reported exposure related to use of protective equipment and findings of biomonitoring studies. Acquavella et al. (2016) highlighted the potential for information bias among case-control studies. Both narrative reviews concluded there was no association between glyphosate and NHL.

The meta-analyses of Schinasi and Leon (2014), Chang and Delzell (2016) and Zhang et al. (2019) evaluated six glyphosate studies, reporting similar meta risk estimates but reaching different conclusions. Schinasi and Leon (2014) reported a statistically significant association of 1.5 (95% CI 1.1–2.0) using univariate estimates. The pooled result from Chang and Delzell was slightly lower (1.3, 95% CI 1.0 – 1.6) with no heterogeneity. Chang and Delzell discussed selection bias, exposure misclassification, and confounding. Using Bradford Hill principles, the authors concluded that the evidence was limited (Chang and Delzell 2016). Zhang et al. (2019) included an updated AHS analysis and pooled results using the *highest* exposure category resulting in a meta risk of 1.41 (95% CI 1.13–1.75), concluding that results “were compelling.”

## Results: toxicology

In the current review of 30 publications, only nine involved some discussion of the animal toxicology literature for an active ingredient (e.g., permethrin, atrazine), with wide variation in the scope and depth of analysis. As shown in Table 2, some authors reviewed the toxicologic data at a high level, while others assessed the associations between pesticide exposure and cancer incidence in laboratory animals, notably with the discussion around mode/mechanism of action and exposure differential between animals and humans. Authors that included some review and discussion of animal carcinogenicity data were fairly consistent in providing definitive conclusions (Table 2) regarding the animal evidence and association with cancer types reported in epidemiologic studies. More specifically, Alavanja et al. (2013), Budnik et al. (2012), Goodman et al. (2015), Jowa et al. (2011), and Von Stackelberg (2013) all reported no evidence from animal studies or no basis for an association between exposure and cancer outcomes for the various specific pesticides they reviewed (Table 2). Boffetta et al. (2018) and Zhang et al. (2019) reported some evidence of tumorigenicity in animals following high exposure levels, which is not uncommon in standard regulatory toxicology testing as it is required that animal be exposed to a range of doses, including what is often termed a maximum tolerated dose. The salient point in such situations is to determine how relevant these exposures are, if at all, to human exposures. Finally, Lewis-Mikhael et al. (2016) and Smith et al. (2017) reported some basis in some test systems for additional investigation regarding carcinogenic outcomes but recommended that further mechanistic work be considered to determine relevance to humans, a point that we have raised earlier and which is now grounded in toxicology interpretation (i.e., the relevance of animal findings to humans).

**Table 2:** Epidemiology studies inclusive of some degree of toxicology review

First author, (Year)	Cancer type	Exposure	Author assessment of animal toxicology data	Conclusion(s) of authors
Alavanja (2013)	Prostate	Terbufos, Malathion, Permethrin, Simazine, Atrazine, Methyl bromide	No direct evidence for prostate cancer	“…new data from toxicology and cancer biology will needed to be used in conjunction with epidemiology to help improve our regulatory procedures and more reliably identify human carcinogens…”
Alavanja (2013)	NHL	2,4-D, MCPA, Glyphosate, Atrazine	No direct evidence for NHL	“…new data from toxicology and cancer biology will needed to be used in conjunction with epidemiology to help improve our regulatory procedures and more reliably identify human carcinogens…”
Boffetta (2018)	Multiple	Permethrin	Animal studies show increased incidence of lung and liver tumors in mice exposed to high doses	“In conclusion, there is no consistent evidence of an increased risk of either liver or lung cancer in humans. Therefore, when the mechanisms of action are taken into consideration, the results of human and animal studies are consistent”
Budnik (2012)	Prostate	Methyl Bromide	No carcinogenic effect following gavage or inhalation exposure	“Carcinogenicity of methyl bromide cannot be easily explained…especially with the limitations and disputed relevance of animal experimentation”
Goodman (2015)	NHL	2,4-D	Lack of association between exposure and cancer outcomes	“3 common modes of action have been proposed including genotoxicity, immunotoxicity, and endocrine/receptor-mediated processes – Taken together, the WOE indicates there is no plausible carcinogenic MOA for 2,4-D…”
Jowa (2011)	Prostate and other cancers	Atrazine	For Fischer rats, all strains of mice and dogs, no evidence of tumors	“Rat-specific hormonal mechanism for mammary tumors now accepted by USEPA, IARC, and EU”
Lewis-Mikhael (2016)	Prostate	Organochlorine pesticides	In vitro prostate cell culture and animal models have provided a basis for studying underlying mechanisms	“…previous mechanisms provide biological plausibility of the studied association; however, their relevance and applicability to carcinogenicity in humans is unknown”
Smith (2017)	NHL	2,4-D	Some studies in rats and mice have shown immunosuppressive effects of 2,4-D, but others have found little or no effect	“Human studies to indicate that this mechanism (immunosuppression) can operate in humans are lacking, however and would be important to perform”
Von Stackelberg (2013)	NHL	2,4-D, MCPA	Toxicology studies in rodents show no evidence of carcinogenicity	“Given that the RfD is a dose associated with no effects and that in vitro bioassay results may not translate to in vivo effects, the combined evidence indicates it is highly implausible that exposures to 2,4-D and/or MCPA are associated with a risk of developing NHL or other lymphohematopoietic cancers”
Zhang (2019)	NHL	Glyphosate-based herbicides	Report inconsistent malignant lymphomas in mice at high dose levels	“The overall evidence from human, animal, and mechanistic studies presented here suggests a compelling link between exposures to GBHs and increased risk for NHL”

The cancer assessments of IARC and the most recent United States Environmental Protection Agency (USEPA) Office of Pesticide Programs cancer risk assessment guidelines provide an in-depth evaluation of specific pesticides and cancer (EPA 2018; Rowland 2006; IARC 2020a). Numerous countries/organizations have similar review systems for carcinogenicity but for brevity, this paper only includes those of USEPA and IARC. Table 3 depicts examples of cancer or tumor type(s) that were associated with the respective active ingredient and then USEPA’s and IARC’s cancer classification for active ingredients based on required lifetime bioassays for evaluation of oncogenicity/cancer in laboratory rats and mice. Note there are differences in classification between the USEPA and IARC due to differing approaches, methods, and types of data reviewed.

**Table 3:** USEPA/IARC cancer classification for active ingredients that have been associated with specific cancer types in epidemiologic studies

Active ingredient	USEPA (EPA 2018; Rowland 2006)	Tumor type and animal species	IARC (2020a)	Cancer type (epidemiology)
Lindane	Suggestive, but not sufficient	Benign lung tumors (female mice only)	1	NHL, prostate
DDT	B2—Probable	Liver—rat/mouse	2A	Breast
Diazinon	Not Likely	Not applicable	2A	NHL
Glyphosate	Not Likely	Not applicable	2A	NHL
Malathion	Suggestive, but not sufficient	Liver (mice), female rats at excessive doses	2A	NHL
2,4-D	D—not classifiable	Not applicable	2B	Multiple, NHL, Prostate
Atrazine	Not likely	Neuroendocrine MOA	3	Multiple, breast, prostate, NHL
Methyl Bromide	Not likely	Not applicable	3	Prostate
Permethrin	Likely	Lung (benign) in females and liver in both sexes of CD1 mice	3	Multiple
MCPA	Not Likely	Not applicable	N/A	Multiple
Terbufos	E—evidence of non-carcinogenicity	Not applicable	N/A	NHL

IARC Cancer Classifications (Group 1: Carcinogenic to humans; Group 2A: Probably carcinogenic to humans; Group 2B: Possibly carcinogenic to humans; Group 3: Not classifiable as to its carcinogenicity to humans), *N/A* not assessed

Under USEPA classification, DDT and permethrin were classified as probable and likely carcinogens, respectively, while other pesticides were “suggestive, but not sufficient,” “not likely” or “with evidence of non-carcinogenicity” based on rodent bioassay data. Additionally, the tumor types in these studies (i.e., liver primarily, although benign lung tumors in female mice exposed to permethrin) were dissimilar from the cancer type(s) reported in epidemiologic studies for these active ingredients (i.e., breast for DDT and multiple types other than liver for permethrin). In comparing across the reported epidemiologic associations between specific pesticide exposure and cancer types (Table 1) from the 30 papers reviewed, there is no animal concordance for the specific cancer/tumor types evaluated in the human studies. This may be due to robust testing requirements for cancer in laboratory animals which typically result in the ability to evaluate carcinogenic outcomes with little ambiguity. While there is no requirement to have site (i.e., same target tissue or organ) concordance when determining human carcinogenic risk from exposure, having site concordance in both animals and humans and then knowing the toxicological mode of action or adverse outcome pathway (i.e., assuming it is the same) for both would strengthen the evidence for an association in humans resulting from exposure. Conversely, if it is determined that the toxicological mode of action in animal studies is not operable or relevant to humans, then this informs the overall evaluation and resultant risk to humans.

## Discussion

The 30 reviews of epidemiologic studies of pesticides and cancer published in the last 10 years highlight the complexity of investigating the risk factors related to farming and other agricultural occupations. Cancers of the lung, breast, and colon were not frequently reviewed and none was associated with a specific pesticide. Prostate cancer and/or NHL were addressed in nearly half of the reviews. Authors reported mixed associations of prostate cancer related to farming and pesticides, in general, with some reviews showing positive associations with farming and not pesticides and vice versa (Krstev and Knutsson 2019; Lewis-Mikahel et al. 2016; Ragin et al. 2013).

Farming and prostate cancer has been a research focal point for many decades (Acquavella et al. 1998; Blair et al. 1992; Keller-Byrne et al. 1997; Van Maele-Fabry and Willems 2004). Generally, the reviews in the last decade characterized results of pesticides and prostate cancer as weak, inadequate, and/or limited to specific groups. For example, an increased risk of certain pesticides and prostate cancer has been reported among farmers with a family history of prostate cancer (Alavanja et al. 2003; Koutros et al. 2013a; Lewis-Mikhael et al. 2016; Silva et al. 2016). While this may reflect a shared environment, the unique nature of farming in which the occupation is passed down through generations and/or a gene-environment interaction, future researchers should consider this among other potential confounders (Koutros et al. 2013b). The findings of a link of prostate cancer with pesticides may be due to poor exposure assessment as suggested by Lewis-Mikhael and colleagues (2016). Studies of specific active ingredients identified a possible association of prostate cancer and methyl bromide exposure but no association with 2,4-D, atrazine or organochlorines.

With respect to NHL, the reviews largely concluded a lack of an association for 2,4-D and atrazine. However, both positive and negative associations were reported for the herbicide glyphosate with reviewers reaching opposing conclusions (Acquavella et al. 2016; Chang and Delzell 2016; Mink et al. 2012; Schinasi and Leon 2014; Zhang et al. 2019). This is also seen among the IARC and governmental regulatory bodies such as the USEPA (Table 3). Potential biases within case–control studies and lack of genotoxic potential were discussed by Acquavella et al. and others (Acquavella et al. 2016; Williams et al. 2016). As discussed above, investigators in the future will be challenged to reduce these limitations to test the hypotheses generated by epidemiologic findings.

Several reviewers opted to use relative risk results for the highest exposed group in each study and incorporated these results into their pooled analysis (Hu et al. 2017; Lewis-Mikhael et al. 2016; Smith et al. 2017; Zhang et al. 2019). While this approach may be seen to provide information in a worst-case scenario, the summary estimates derived are only meaningful for situations in which the underlying studies use homogeneous exposure categories. In the papers evaluated here, this was not always the case. For example, in the Smith meta-analysis for NHL and “highest” 2,4-D exposure, the exposure metric varied widely across the individual studies (Smith et al. 2017). The exposure proxy, “duration of use,” was defined as seven days (McDuffie et al. 2001), 21 days (Zahm et al. 1990), five years (Burns et al. 2011) and 10 years (Kogevinas et al. 1995) in four studies. However, these widely varying definitions of high exposure were considered to be equivalent in the meta-analysis. In other words, the reviewers created a risk estimate for “highly exposed groups” that implied a homogeneity that did not exist. Future reviews that seek to combine information from two or more studies would be strengthened if investigators utilized exposure categories that were concordant with those previously reported (Burns et al. 2019).

A review of the animal toxicology data for pesticides associated with cancer types in the epidemiologic reviews (i.e., in those reviews in which animal toxicity data were reviewed and discussed) indicated little biological plausibility or empirical data in support of an association between exposure and cancer in humans. Encouragingly, among those reviews which evaluated animal toxicology data, many discussed or recommended that advancements in toxicological research (e.g., mode of action) be considered or applied when evaluating epidemiological data -in this case relative to cancer association or causation in humans from pesticidal exposure. Alavanja, et al. recommended that “new data from toxicology and cancer biology will need to be used in conjunction with epidemiology to help improve our regulatory procedures and more reliably identify human carcinogens…”(Alavanja et al. 2013). In addition, it is notable that 6 of the 9 authors that reviewed toxicology data in conjunction with epidemiological data pointed to the consideration and use of mechanistic data on different levels (i.e., in vitro, receptor-mediated, in vivo animal) to assist in interpreting the relevance and applicability of animal data for humans (Table 2).

Evaluation and integration of data across the epidemiology and toxicology disciplines can be challenging (e.g. (Adami et al. 2011; Rhomberg 2015)), but it has been discussed within the scientific and regulatory communities as helpful in establishing biological plausibility and causal inference (Acquavella et al. 2003; Adami et al. 2011; Peklonen et al. 2019). Review organizations such as IARC and regulatory agencies compare and contrast existing animal toxicology data, including mode of action and its relevance to humans, in discussions involving associations between pesticide exposure and cancer outcomes in humans. This is important as (1) global regulatory bodies or organizations (i.e., USEPA, EFSA) are increasingly assessing both data streams (i.e., epidemiology and toxicology) for risk assessment and cancer classification and (2) contemporary knowledge on cancer biology and etiology continues to expand (Cohen et al. 2019; Doe et al. 2019; Wolf et al. 2019), which informs the ability to identify relationships between chemical exposure and association with cancer outcomes in humans. In addition, a particularly important consideration in the evaluation of reported human health outcomes from pesticide exposure is the exposure characterization across studies (i.e., animals and humans). While precise exposure estimates are rarely available in human research as they are in animal studies, efforts to quantify biomonitoring data in a risk-based context is informative. For example, urinary concentrations of 2,4-D among farmers and applicators have been shown to be lower than the regulatory reference dose (Aylward and Hays 2015). Acquavella, et al. recommended that epidemiologists consider the regulatory reference dose and No Observed Adverse Effect Levels (NOAEL) of pesticides when collecting exposure data (Acquavella et al. 2003). Epidemiologic research by job class and/or occupational group is hampered by the diversity of exposures to individuals. Going forward, investigators will need creative and novel approaches to better capture specific and multiple exposures, temporal changes, and use of personal protection equipment (Beane Freeman 2020). It is hoped that future epidemiologic investigations can improve and refine the quantification of exposure to facilitate data integration.

While humans are not rodents, it is advisable to evaluate the predictability and accuracy of rodent oncogenicity studies for humans. Animal testing affords insight into a chemical’s carcinogenic potential, yields insight on the mode of action (or adverse outcome pathway) for that chemical, and informs on dose–response related to tumor response and how those exposures compare to human exposures. Animal models are not perfect predictors of toxicological or carcinogenic response in humans, but advancements in toxicology and exposure science have further progressed the ability to better predict outcomes in humans. Toxicological testing, evaluation and risk assessment programs such as those of the USEPA rigorously strive to protect human health by controlling and regulating human exposure to pesticides as mandated by law. These assessments and the subsequent limits on human exposure occur whether or not rodent tests show carcinogenic responses and the mode of action and exposures known to be associated with oncogenic response in rodents are relevant to humans (EPA 2002, 2005).

Relative to advancements in toxicology testing and how they are integrated into an overall approach for the protection of human health, including consideration of human epidemiologic studies, it has been proposed to move from the 2-year cancer bioassay in animals to a decision-tree matrix. The latter presumes that cancer is the consequence of DNA coding errors, arising from either direct mutagenic events or indirect sustained cellular proliferation (Cohen et al. 2019). Assessment of mutagenic (i.e., DNA reactive) activity through in vitro and in silico evaluations allows determination of mutagenic activity. If mutagenic, the chemical is assumed to be carcinogenic unless evidence indicates otherwise—if the chemical does not show mutagenic potential, an assessment of potential human exposure is compared to the threshold for toxicological concern. If anticipated exposure exceeds the threshold, then evaluations are conducted to look for key precursors to carcinogenicity such as increased cell proliferation, immunosuppression or significant estrogenic activity. Protection of human health is then approached by limiting exposure to below the established NOAELs for these precursor events. Moving forward, these advancements in toxicity testing, coupled with a novel and refined approaches for evaluating human exposures in epidemiologic studies, should afford more accurate and insightful learnings about associations between pesticide use and human disease, including cancer.

## Conclusion

Just as cancer is not a unique disease, the exposures to “pesticides” and “farming” are similarly diverse. Interpretations are most informative when assessed by specific cancer type and active ingredients. However, it is a well-known challenge to collect these data reliably and validly with an adequate sample size, oftentimes retrospectively, all of which limits the ability to compare and contrast those exposures used in toxicology studies. For the last decade, there has been attention on the consideration, if not integration, of both animal and epidemiologic data for both risk assessment and regulatory decision-making [e.g. (Adami et al. 2011; Christensen et al. 2015)] and this remains a priority going forward. This review sought to compare the human and animal data relative to specific cancer outcomes reported in epidemiologic studies. While several pesticidal active ingredients were associated with specific cancer, overall, there was neither strong nor consistent epidemiologic data supportive of a positive association between pesticide exposure in occupational settings and cancer. In recent years, there have been progressive, science-based approaches for determining the oncogenic potential from chemicals, including pesticides, which include identification of genotoxic potential, toxicological mode of action, relevance to humans, and assessment of human exposure relative to doses used in animal experimentation. In addition, epidemiologic studies offer critical information and insight on exposure and disease, including cancer in humans. The challenge and opportunity at hand are for enhanced multidisciplinary collaboration to understand the contributions and limitations of both toxicologic and epidemiologic research so that underpinning scientific questions and informed study design will increase the ability and confidence to inform and protect human health.

## Supplementary Information

Below is the link to the electronic supplementary material.

Supplementary file1 (DOCX 53 KB)
