# Supplementary material for: Cancer and occupational exposure to pesticides: an umbrella review
Source: Int Arch Occup Environ Health. 2021 Jan 25;94(5):945–57. doi: 10.1007/s00420-020-01638-y (PMC8238729; doi:10.1007/s00420-020-01638-y)
Supplement: Supplementary file 1 — Supplementary file1 (DOCX 53 KB) [file 420_2020_1638_MOESM1_ESM.docx]

Appendix Table A1. Reviews of occupational studies of prostate cancer

| **First Author, year**  **[Ref #]** | **Exposure** | **Narrative vs. Quantitative** | **Meta-analysis results (95% CI)** | **I^2^ (number of studies)** | **Conclusion(s) of Authors** |
| --- | --- | --- | --- | --- | --- |
| Alavanja, 2012  [1] | Sub-results on multiple specific actives | Narrative | N/A | (13 studies*) | *“…a number of specific pesticides have been linked to prostate cancer…” …” risk seems to be enhanced by a family history of prostate cancer.”* |
| Boffetta, 2013  [2] | Atrazine | Narrative | N/A | (6 studies) | *"…do not support a causal relationship"* |
| Budnik, 2012 [3] | Methyl bromide | Quantitative | MB: mOR = 1.21 (0.98 – 1.49) | 0% (3 studies) | *“…suggest a possible link”* |
| Depczynski, 2014 [4] | Pesticides, farming | Narrative | N/A | (18 studies) | *“…overall evidence for increased prostate cancer risk in farmers was weak.”* |
| Doolan, 2014 [5] | Chemical Exposures that include studies of pesticides and/or farming | Narrative | N/A | (30 studies*) | *“…risk levels were too low to significantly implicate pesticides…”* |
| Goodman, 2015 [6] | 2,4-D | Quantitative | 2,4-D: mRR = 1.32 (0.37 – 4.69) | 87.0% (2 studies) | *“2,4-D was not associated with … prostate cancer.”* |
| Krstev, 2019 [7] | Pesticides, farming | Quantitative | Pesticides: mRR = 1.15 (1.01 – 1.32)  Farming: mRR = 0.99 (0.95 – 1.02)  OC: mRR = 1.08 (1.03 – 1.14),  OP: mRR = 0.98 (0.87 – 1.11),  Carbamates: mRR = 1.05 (0.89 – 1.24)  Triazines: mRR = 1.02 (0.92 – 1.14) | 84% (18 studies)  79%, (26 studies)  0% (17 studies)  0% (7 studies)  27% (5 studies)  45% (4 studies) | *“Our meta-analysis showed a statistically significant excess risk of prostate cancer…”no evidence that farm work was associated with increased risk of prostate cancer.”* |
| Jowa, 2011 [8] | Atrazine (& related chlorotriazines) | Narrative | N/A | (8 studies*) | *“there is inadequate evidence at this time to conclude that exposure to atrazine or simazine leads to a higher risk of…prostate cancer”.* |
| Lewis-  Mikhael, 2016 [9] | Pesticides | Quantitative | Ever used pesticides: mOR = 1.27 (0.92 – 1.63)  Ever used pesticides: mRR = 1.27 (0.65 – 1.89)  High quality (NOS): mOR = 0.88 (0.53 – 1.23)  OC: mOR = 1.35 (1.02 – 1.67), | 54.3% (21 case control studies)  95.5% (4 cohort studies)  34.2%, (4 studies)  0% (8 studies) | *“…positive findings were mostly confined to farmers exposed to high levels of specific groups of pesticides.” …Our results suggest that less accuracy of pesticide exposure assessment exaggerated the magnitude of the association with [prostate cancer].* |
| Lewis-Mikhael, 2015 [10] | Specific organochlorine | Quantitative | All based upon “high” exposure  DDT: mOR = 1.14 (0.81 – 1.47),  DDE: mOR = 1.02 (0.69 – 1.35),  Heptachlor: mOR = 0.95 (0.25 – 1.66)  Hexachlorobenzene: mOR = 0.88 (0.18 – 1.57)  Oxychlordane: mOR = 0.91 (0.46 – 1.35),  Trans-nonachlor: mOR = 0.88 (0.45 – 1.31)  Lindane: mOR = 1.56, (0.82 – 2.29) | 30.2% (5 studies)  12.7% (5 studies)  79.1% (3 studies)  36.0% (3 studies)  0% (4 studies)  0% (3 studies)  41.7% (3 studies). | *“…no concrete evidence of an association between specific OCPs and [prostate cancer]”* |
| Ntzani, 2013 [11] | Sub-results on multiple specific actives | Narrative | N/A | (39 studies) | *“Overall, there is no evidence supporting an association between pesticide exposure and prostate cancer.”* |
| Ragin, 2013 [12] | Pesticides, farming | Quantitative | Farmers: mOR= 3.83 (1.96 – 7.48)  Farmers: mOR = 1.38 (1.16 – 1.64  Pesticide use: mOR = 0.74 (0.40 – 1.34), | (2 studies using benign prostate hyperplasia controls)  31% (5 studies using non-benign prostate hyperplasia controls)  68.9% (4 studies) | *“…farming is associated with an increased risk of prostate cancer,” …” pesticide exposure among farmers does not appear to significantly contribute to prostate cancer risk.”* |
| Sathiakumar, 2011 [13] | Triazines (esp atrazine) | Narrative | N/A | (8 studies*) | *“Collectively, the available epidemiology studies do not provide consistent, scientifically convincing evidence of a causal relationship”* |
| Silva, 2016 [14] | Pesticides | Narrative | N/A | (49 studies) | *“…pesticide exposure is associated with a moderate increase in the risk of developing prostate cancer.”* |

*Based on a count of the studies, number not reported by the authors; OC: Organochlorine, OP: Organophosphate

Appendix Table A2. Reviews of occupational studies of non-Hodgkin lymphoma (NHL)

| **First Author, year**  **[Ref #]** | **Exposure** | **Narrative vs. quantitative** | **Meta-analysis results (95% CI)** | **I^2^ (number of studies)** | **Conclusion(s) of Authors** |
| --- | --- | --- | --- | --- | --- |
| Acquavella, 2016 [15] | Glyphosate | Narrative | N/A | (7 studies) | *“This study found no evidence of an association between glyphosate and NHL”* |
| Alavanja, 2012 [1] | Sub-results on multiple specific actives | Narrative | N/A | (21 studies*) | *“epidemiological evidence for certain pesticides and NHL is growing” …” Few studies of pesticides have been large enough to evaluate the potential link between NHL subtypes and specific pesticide exposures.”* |
| Boffetta, 2013 [2] | Atrazine | Narrative | N/A | (5 studies) | *“Lack of a causal association between atrazine and NHL”* |
| Burns, 2012 [16] | 2,4-D | Narrative | N/A | (16 studies) | *“largest, most robust studies found no dose response and no statistically significant increase in use of 2,4-D and NHL.”*  *“Overall, there are a few statistically significant positive observations, but the data are not consistent across studies, particularly in the past 10 years.”* |
| Chang, 2016 [17] | Glyphosate | Quantitative | mRR = 1.3 (1.0 – 1.6) | 0% (6 studies) | *“…no causal relationship has been established between glyphosate exposure and risk of NHL…or any subtype of LHC”* |
| Goodman, 2015 [6] | 2,4-D | Quantitative | mRR = 0.97 (0.77 – 1.22) | 28.8% (9 studies) | *“Does not support an association…”* |
| Goodman, 2017 [18] | 2,4-D | Quantitative | mRR = 0.97 (0.79 – 1.18) | 20.4% (10 studies) | *“Growing body…that 2,4-D does not cause cancer in humans”* |
| Hu, 2017 [19] | OP (terbufos, malathion, diazinon) | Quantitative | Malathion: mOR = 1.17 (0.82 – 1.67)  Diazinon: mOR = 1.39 (1.11 – 1.73)  Terbufos: mOR = 1.07 (0.85 – 1.36), | 73.2% (7 studies)  0% (7 studies)  0%, (5 studies) | *“…Terbufos …and Malathion had a statistically non-significant relationship, whereas Diazinon … was significantly associated with an increased NHL risk.”* |
| Jayakody, 2015 [20] | Phenoxy herbicides ** | Narrative | N/A | (32 studies) | *“…difficulties in discriminating small relative risks for rare health outcomes from exposures that are also fairly uncommon”* |
| Jowa, 2011 [8] | Atrazine (& related chlorotriazines) | Narrative | N/A | (10 studies*) | *there is inadequate evidence at this time to conclude that exposure to atrazine or simazine leads to a higher risk of…non-Hodgkin’s lymphoma””.* |
| Mink, 2012 [21] | Glyphosate | Narrative | N/A | (8 studies) | *“…cautious interpretation of the few positive associations reported and conclude that the epidemiologic data, considered together do not support a causal association between glyphosate exposure and cancer”* |
| Ntzani, 2013 [11] | Sub-results on multiple specific actives) | Narrative | N/A | (44 studies) | *“results need adjustment for multiple testing.* |
| Sathiakumar, 2011 [13] | Triazine herbicides, esp atrazine | Narrative | N/A | (10 studies*) | *“Collectively, the available epidemiology studies do not provide consistent, scientifically convincing evidence of a causal relationship…”* |
| Schinasi, 2014 [22] | sub-results on multiple specific actives | Quantitative | 2,4-D: mRR = 1.4 (1.0 – 1.9)  Glyphosate: mRR = 1.5 (1.1 – 2.0)  Triazine: mRR = 1.5 (1.0 – 2.1)  Carbamates: mRR = 1.7 (1.3 – 2.3)  OP: mRR = 1.6 (1.4 – 1.9)  Lindane: mRR = 1.6 (1.2 – 2.2) | 61.5%) (5 studies)  32.7% (6 studies)  38.5% (4 studies)  0% (3 studies)  0%, (4 studies)  26.0% (4 studies) | *“positive associations between NHL and carbamate insecticides, organophosphorous insecticides, lindane…and MCPA.”* |
| Smith, 2017 [23] | 2,4-D | Quantitative | Highest: mRR=1.38 (1.10 – 1.73)  Ever vs never: mRR = 1.31 (1.13 – 1.52), | 56% (12 studies)  50% (12 studies) | *"…highest exposure group… we identified a statistically significant association between 2,4-D exposure and increased RRs of NHL.”* |
| Von Stackelberg, 2013 [24] | 2,4-D, MCPA | Narrative | N/A | (41 studies*) | *“Show inconsistent relationships between exposure to chlorophenoxy compounds generally, 2,4-D and/or MCPA specifically, and lymphohematopoietic outcomes.”* |
| Zhang, 2019 [25] | Glyphosate | Quantitative | Highest: mRR = 1.41 (1.13 – 1.75) | 39.4% (6 studies) | *“…suggests a compelling link between exposures to GBHs and increased risk for NHL.”* |

* Based on a count of the studies, number not reported by the authors; ** Phenoxy herbicides included 2,4-D, 2,4,5-T, 2,4 DP, MCPA, MCPP.; OP: organophosphate

References for Tables A1 and A2

1. Alavanja MC, Bonner MR (2012) Occupational pesticide exposures and cancer risk: a review. J Toxicol Environ Health B Crit Rev 15 (4):238-263. doi:10.1080/10937404.2012.632358

2. Boffetta P, Adami HO, Berry SC, Mandel JS (2013) Atrazine and cancer: a review of the epidemiologic evidence. European journal of cancer prevention : the official journal of the European Cancer Prevention Organisation (ECP) 22 (2):169-180. doi:10.1097/CEJ.0b013e32835849ca

3. Budnik LT, Kloth S, Velasco-Garrido M, Baur X (2012) Prostate cancer and toxicity from critical use exemptions of methyl bromide: Environmental protection helps protect against human health risks. Environ Health 11. doi:10.1186/1476-069x-11-5

4. Depczynski J, Lower T (2014) A review of prostate cancer incidence and mortality studies of farmers and non-farmers, 2002–2013. Cancer Epidemiol 38 (6):654-662. doi:<https://doi.org/10.1016/j.canep.2014.09.001>

5. Doolan G, Benke G, Giles G (2014) An Update on Occupation and Prostate Cancer. Asian Pac J Cancer Prev 15 (2):501-516. doi:10.7314/apjcp.2014.15.2.501

6. Goodman JE, Loftus CT, Zu K (2015) 2,4-Dichlorophenoxyacetic acid and non-Hodgkin's lymphoma, gastric cancer, and prostate cancer: meta-analyses of the published literature. Ann Epidemiol 25 (8):626-636. doi:10.1016/j.annepidem.2015.04.002

7. Krstev S, Knutsson A (2019) Occupational Risk Factors for Prostate Cancer: A Meta-analysis. J Cancer Prev 24 (2):91-111. doi:10.15430/jcp.2019.24.2.91

8. Jowa L, Howd R (2011) Should atrazine and related chlorotriazines be considered carcinogenic for human health risk assessment? J Environ Sci Health C Environ Carcinog Ecotoxicol Rev 29 (2):91-144. doi:10.1080/10590501.2011.577681

9. Lewis-Mikhael AM, Bueno-Cavanillas A, Ofir Giron T, Olmedo-Requena R, Delgado-Rodriguez M, Jimenez-Moleon JJ (2016) Occupational exposure to pesticides and prostate cancer: a systematic review and meta-analysis. Occup Environ Med 73 (2):134-144. doi:10.1136/oemed-2014-102692

10. Lewis-Mikhael AM, Olmedo-Requena R, Martinez-Ruiz V, Bueno-Cavanillas A, Jimenez-Moleon JJ (2015) Organochlorine pesticides and prostate cancer, Is there an association? A meta-analysis of epidemiological evidence. Cancer Causes Control 26 (10):1375-1392. doi:10.1007/s10552-015-0643-z

11. Ntzani EE, Ntritsos G, M C, Evangelou E, Tzoulaki I (2013) Literature review on epidemiological studies linking exposure to pesticides and health effects. EFSA Supporting Publications 10 (10):159 pp. doi:10.2903/sp.efsa.2013.EN-497

12. Ragin C, Davis-Reyes B, Tadesse H, Daniels D, Bunker CH, Jackson M, Ferguson TS, Patrick AL, Tulloch-Reid MK, Taioli E (2013) Farming, Reported Pesticide Use, and Prostate Cancer. Am J Mens Health 7 (2):102-109. doi:10.1177/1557988312458792

13. Sathiakumar N, MacLennan PA, Mandel J, Delzell E (2011) A review of epidemiologic studies of triazine herbicides and cancer. Crit Rev Toxicol 41:1-34. doi:10.3109/10408444.2011.554793

14. Silva JF, Mattos IE, Luz LL, Carmo CN, Aydos RD (2016) Exposure to pesticides and prostate cancer: systematic review of the literature. Rev Environ Health 31 (3):311-327. doi:10.1515/reveh-2016-0001

15. Acquavella J, Garabrant D, Marsh G, Sorahan T, Weed DL (2016) Glyphosate epidemiology expert panel review: a weight of evidence systematic review of the relationship between glyphosate exposure and non-Hodgkin's lymphoma or multiple myeloma. Crit Rev Toxicol 46:28-43. doi:10.1080/10408444.2016.1214681

16. Burns CJ, Swaen GM (2012) Review of 2,4-dichlorophenoxyacetic acid (2,4-D) biomonitoring and epidemiology. Crit Rev Toxicol 42 (9):768-786. doi:10.3109/10408444.2012.710576

17. Chang ET, Delzell E (2016) Systematic review and meta-analysis of glyphosate exposure and risk of lymphohematopoietic cancers. J Environ Sci Health B 51 (6):402-428. doi:10.1080/03601234.2016.1142748

18. Goodman JE, Loftus CT, Zu K (2017) 2,4-Dichlorophenoxyacetic acid and non-Hodgkin's lymphoma: results from the Agricultural Health Study and an updated meta-analysis. Ann Epidemiol 27 (4):290-292.e295. doi:10.1016/j.annepidem.2017.01.008

19. Hu L, Luo D, Zhou T, Tao Y, Feng J, Mei S (2017) The association between non-Hodgkin lymphoma and organophosphate pesticides exposure: A meta-analysis. Environmental pollution (Barking, Essex : 1987) 231 (Pt 1):319-328. doi:10.1016/j.envpol.2017.08.028

20. Jayakody N, Harris EC, Coggon D (2015) Phenoxy herbicides, soft-tissue sarcoma and non-Hodgkin lymphoma: a systematic review of evidence from cohort and case-control studies. Br Med Bull 114 (1):75-94. doi:10.1093/bmb/ldv008

21. Mink PJ, Mandel JS, Sceurman BK, Lundin JI (2012) Epidemiologic studies of glyphosate and cancer: A review. Reg Toxicol Pharm 63 (3):440-452. doi:10.1016/j.yrtph.2012.05.012

22. Schinasi L, Leon ME (2014) Non-Hodgkin lymphoma and occupational exposure to agricultural pesticide chemical groups and active ingredients: a systematic review and meta-analysis. Int J Environ Res Public Health 11 (4):4449-4527. doi:10.3390/ijerph110404449

23. Smith AM, Smith MT, La Merrill MA, Liaw J, Steinmaus C (2017) 2,4-dichlorophenoxyacetic acid (2,4-D) and risk of non-Hodgkin lymphoma: a meta-analysis accounting for exposure levels. Ann Epidemiol 27 (4):281-289. doi:10.1016/j.annepidem.2017.03.003

24. von Stackelberg K (2013) A Systematic Review of Carcinogenic Outcomes and Potential Mechanisms from Exposure to 2,4-D and MCPA in the Environment. J Toxicol. doi:10.1155/2013/371610

25. Zhang L, Rana I, Shaffer RM, Taioli E, Sheppard L (2019) Exposure to glyphosate-based herbicides and risk for non-Hodgkin lymphoma: A meta-analysis and supporting evidence. Mutation Research-Reviews in Mutation Research 781:186-206. doi:10.1016/j.mrrev.2019.02.001
